# Rapidly Enlarging Forearm Nodule Representing Keratoacanthoma-Type Crateriform Squamous Cell Carcinoma in a 54-Year-Old Man: A Case Report

**DOI:** 10.7759/cureus.89039

**Published:** 2025-07-30

**Authors:** Ahmad Y Karim, Krithika Giresh, Michael Vanderloo

**Affiliations:** 1 Trauma Surgery, William Carey University College of Osteopathic Medicine, Hattiesburg, USA; 2 Family Medicine, William Carey University College of Osteopathic Medicine, Hattiesburg, USA; 3 Family Medicine, Forrest General Hospital, Hattiesburg, USA

**Keywords:** crateriform architecture, cutaneous squamous cell carcinoma, forearm lesion, histopathology, immunohistochemistry, keratoacanthoma, keratoacanthoma‑type squamous cell carcinoma, mohs micrographic surgery, sun‑exposed skin, wide local excision

## Abstract

Keratoacanthoma-type crateriform squamous cell carcinoma is an uncommon cutaneous neoplasm that clinically mimics benign keratoacanthoma yet carries malignant potential. This case report describes a 54-year-old Caucasian man with occupational sun exposure and no past medical or significant family history, who developed a rapidly enlarging, dome-shaped nodule with a central keratin plug on his left forearm over six weeks. The lesion was removed via full-thickness fusiform excision with 4 mm margins and closed primarily; histopathology confirmed a crateriform squamous cell carcinoma with clear margins. The surgical site healed uneventfully, and the patient remains disease-free at the three-month follow-up. This report highlights the key clinical and histopathological features that distinguish this variant, reviews optimal surgical management and the need for ongoing surveillance, and aims to enrich the limited literature on keratoacanthoma-type crateriform squamous cell carcinoma to aid clinicians in prompt recognition and treatment.

## Introduction

Keratoacanthoma is a crateriform epithelial tumor defined by a symmetrical, cup‑shaped epidermal invagination lined by well‑differentiated keratinocytes, capped by a keratin plug, and supported by a broad "pushing" base [1]. It occurs predominantly on chronically sun-exposed skin of middle-aged and older adults, with a male-to-female ratio of about 2:1 and a characteristic rapid growth over weeks to a 1-2 cm dome-shaped nodule that may bleed or become tender [2]. Although spontaneous regression is described in a proportion of cases, shared molecular features with cutaneous squamous cell carcinoma (cSCC), most notably TP53 (≈30-50%) and RAS (≈20-30%) mutations, support malignant potential and the need for timely treatment [3,4]. The clinical differential includes well‑differentiated (crateriform) cSCC, hypertrophic actinic keratosis, cutaneous horn, pseudoepitheliomatous hyperplasia, nodular/ulcerated basal cell carcinoma, and infectious or reactive keratinizing lesions, making histopathology the diagnostic standard [2,4]. When morphology is equivocal, immunohistochemistry can assist: aberrant p16 and p53 with a diffuse Ki‑67 pattern favor cSCC, whereas keratoacanthoma more often shows a peripheral, graded Ki‑67 pattern [5]. Consistent with cSCC guidance, complete excision with 3-5 mm clinical margins is recommended for diagnosis and cure, with Mohs micrographic surgery reserved for recurrent or anatomically sensitive sites; curettage-electrodesiccation and intralesional methotrexate (MTX) or 5‑FU are options when surgery must be minimized or contraindicated [6-8]. Crateriform lesions can cause local tissue destruction and rarely behave aggressively, and post‑treatment surveillance aligned with cSCC practices is prudent to detect recurrence or high‑risk features (e.g., perineural invasion) early [2,9].

## Case presentation

**Figure 1:**
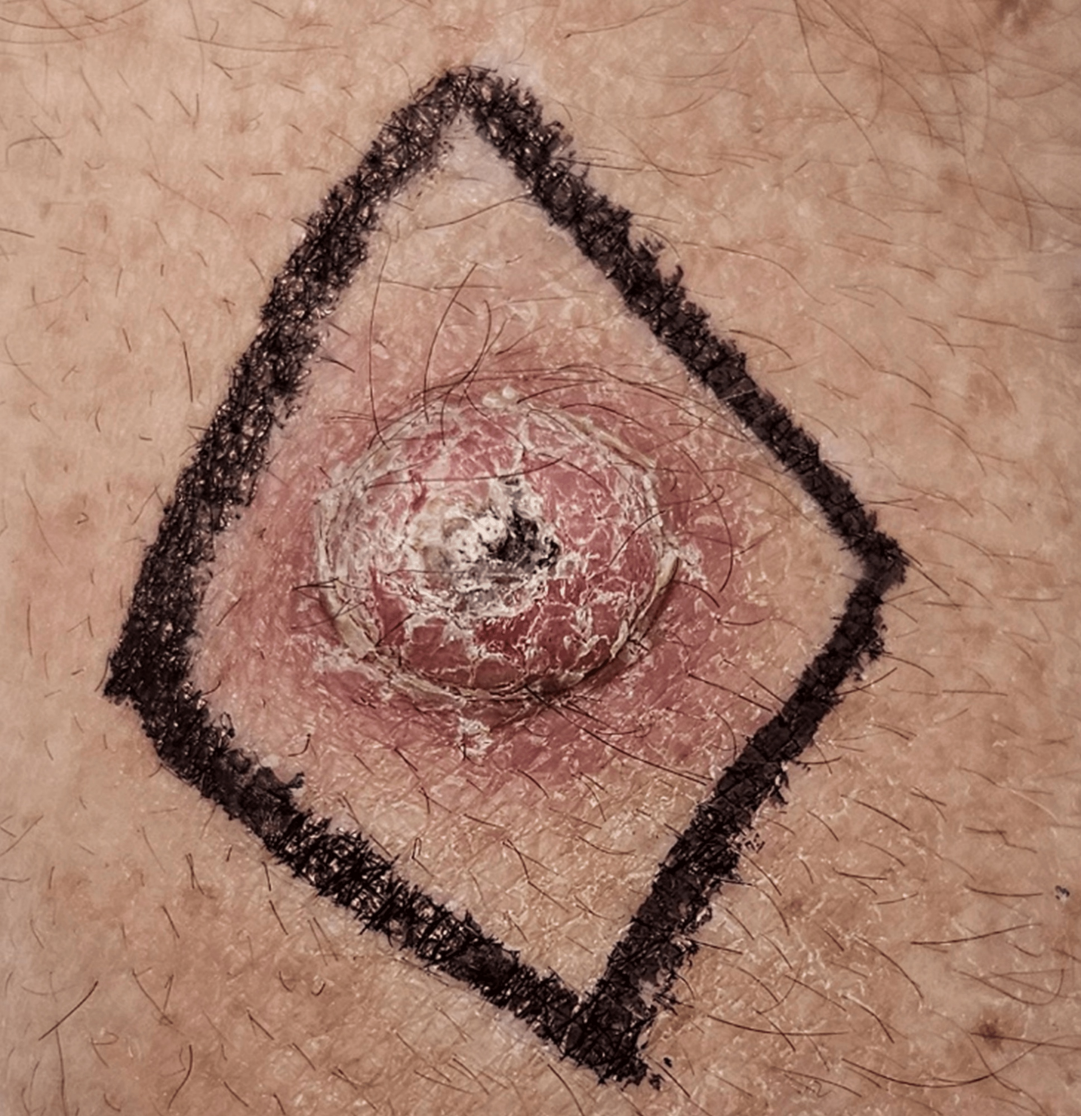
Pre‑excision clinical photograph of keratoacanthoma‑type crateriform squamous cell carcinoma on the left forearm Pre‑excision clinical image of the left dorsal forearm showing a dome‑shaped, erythematous nodule with a central keratin plug and overhanging epidermal lip, classic for keratoacanthoma‑type crateriform squamous cell carcinoma.

A 54-year-old Caucasian man with occupational sun exposure and no past medical or significant family history noticed a painless "pimple-sized" papule on the dorsal left forearm. Over six weeks, the lesion enlarged to 14 mm, became mildly tender, and bled intermittently. Examination revealed a dome-shaped erythematous nodule with a central keratin plug and an overhanging epidermal rim (Figure 1). 

**Figure 2:**
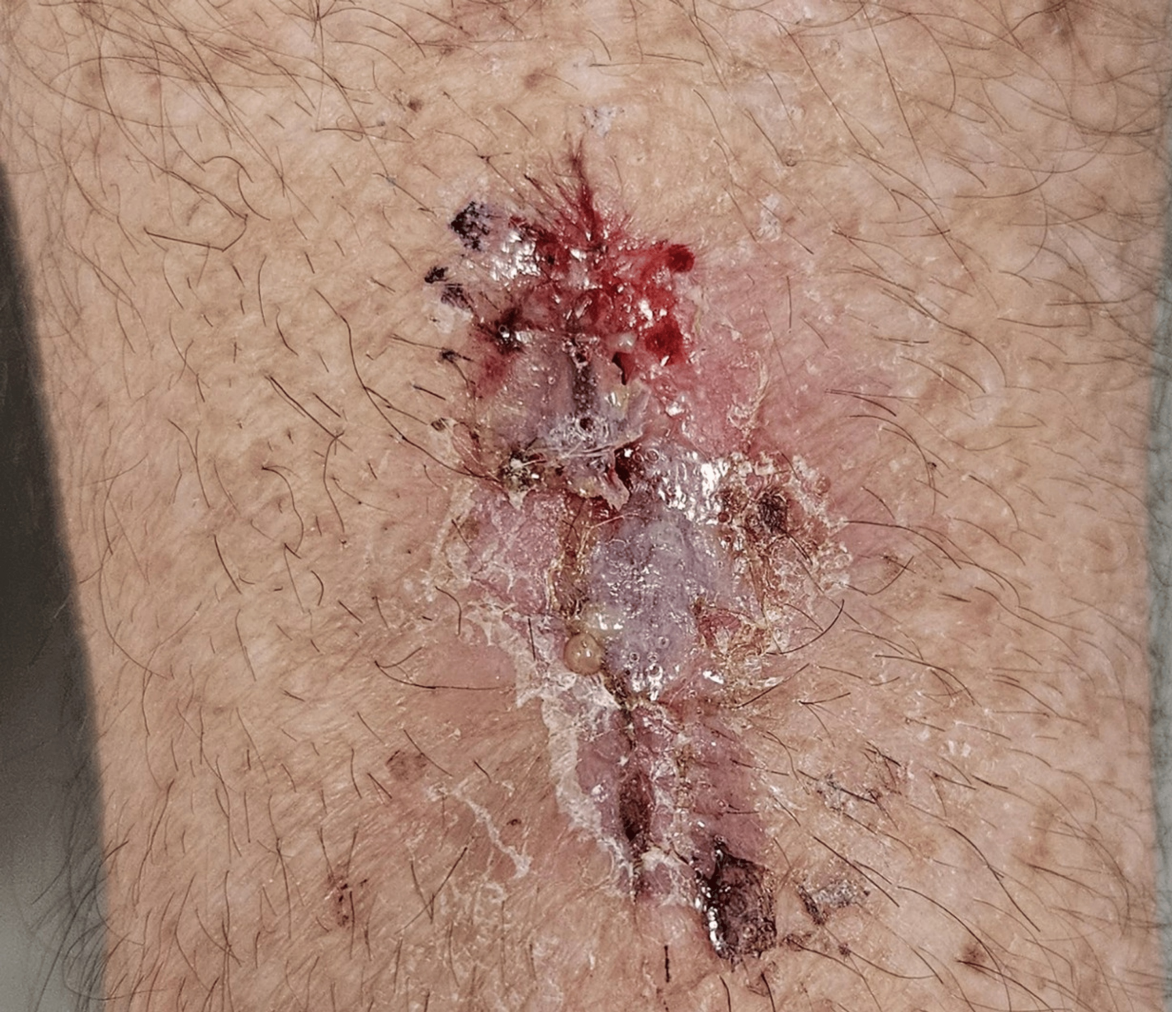
Ten‑day post‑excision clinical photograph of forearm incision Clinical image of the left dorsal forearm 10 days after fusiform excision and primary closure. The incision is fully epithelialized, exhibiting a thin hemorrhagic crust and mild peri‑incisional erythema, consistent with uncomplicated wound healing following 4‑0 polyglactin closure.

No regional lymphadenopathy was detected. Under local anesthetic, a fusiform excision (25×20×10 mm) with 4 mm peripheral margins and subcutaneous depth was performed. The specimen was oriented with sutures and submitted in formalin. The wound was closed with buried 4-0 polyglactin. The dressings were removed after 48 hours, and the patient resumed light duties on day 3. Sutures were removed on day 10; the incision showed complete epithelialization with a thin hemorrhagic crust (Figure 2).

At the three-month follow-up, the scar was flat and supple, and there was no evidence of recurrence.

**Figure 3:**
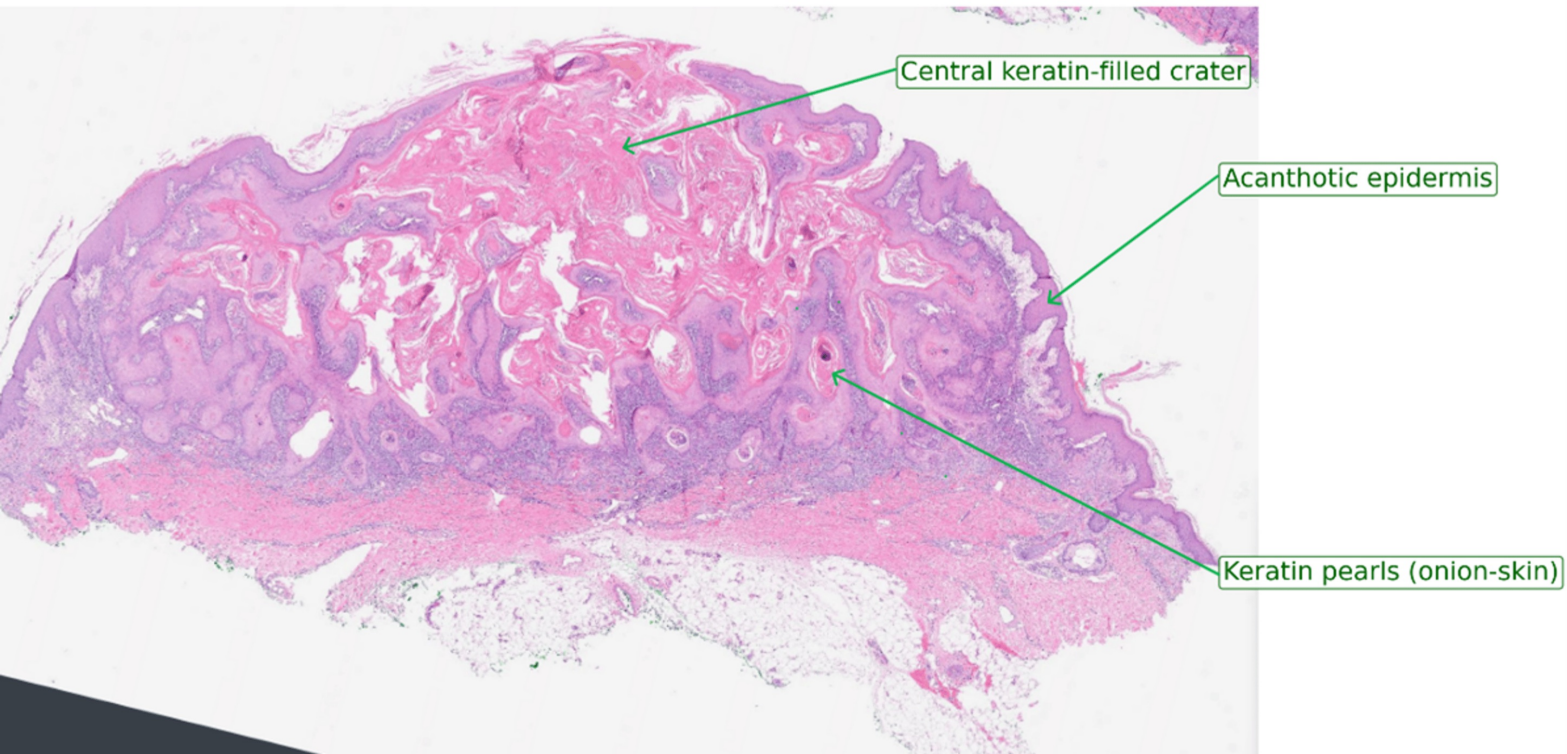
Low‑power H&E of keratoacanthoma‑type crateriform squamous cell carcinoma H&E-stained low‑power view of the excised forearm lesion, demonstrating a symmetrical, cup‑shaped invagination filled with laminated keratin (central keratin‑filled crater), an overhanging acanthotic epidermal rim, and underlying well‑differentiated squamous nests with concentric keratin pearls ("onion‑skinning"). Scale bar=2 mm.

The low-power H&E sections revealed a symmetrical cup-shaped crater filled with laminated keratin and flanked by markedly acanthotic epidermis (Figure 3).

**Figure 4:**
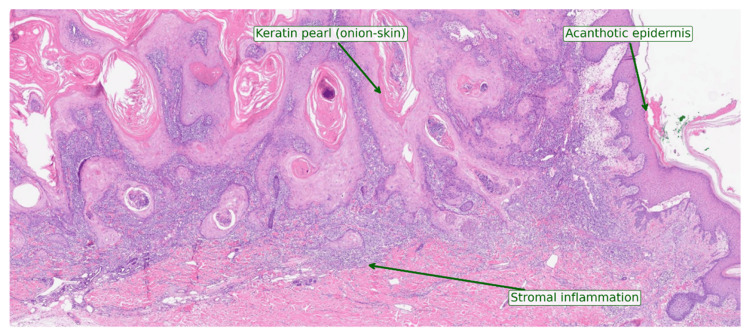
High‑power H&E (×100) of keratoacanthoma‑type crateriform squamous cell carcinoma H&E-stained section at ×100 magnification showing well‑differentiated squamous nests with concentric keratin pearls (arrow), overlying acanthotic epidermis (arrowhead), and a dense mixed stromal inflammatory infiltrate (asterisk). The squamous cells exhibit only mild cytologic atypia, consistent with keratoacanthoma‑type crateriform squamous cell carcinoma.

High-power view showed well-differentiated squamous nests with concentric keratin pearls, mild pleomorphism, and a dense mixed inflammatory infiltrate; no perineural or lymphovascular invasion was present (Figure 4).

All margins were tumor-free by ≥3 mm. The final diagnosis was keratoacanthoma-type crateriform squamous cell carcinoma.

## Discussion

The presentation in our male patient in his sixth decade with a rapidly enlarging, keratin‑plugged nodule on a sun‑exposed forearm matches the typical age, sex, and anatomic distribution of solitary keratoacanthoma reported in reviews and guidelines [2]. Management by complete excision with 3-5 mm margins aligns with guideline‑concordant care for keratoacanthoma‑type lesions and low‑risk cSCC [6,9]. Published series indicate high clearance with standard excision, whereas nonsurgical approaches are reserved for select scenarios: in a 157‑tumor cohort, intralesional MTX achieved high clearance on its own and reached 100% when non‑responders underwent delayed excision [7]; a randomized trial showed neoadjuvant MTX significantly reduced lesion size and facilitated smaller, cosmetically favorable excisions [8]. These data contextualize our short‑term outcome (uneventful healing, no recurrence at three months) as in keeping with expected results after complete excision while reinforcing the need for ongoing surveillance because crateriform cSCC can rarely recur or show aggressive features such as perineural invasion [2,6,9]. Table 1 summarizes distinguishing clinicopathologic features of keratoacanthoma versus cSCC (histology and marker patterns derived from [1,5]).

**Table 1:** Comparison of keratoacanthoma vs. cutaneous squamous cell carcinoma

Feature	Keratoacanthoma	Cutaneous squamous cell carcinoma
Growth pattern	Rapid over weeks	Gradual over months
Regression	Common (>60%)	Rare
Histology	Symmetrical crater, pushing borders	Irregular, infiltrative borders
Mutations	TP53 (30-50%)	TP53, RAS, NOTCH1, CDKN2A
	RAS (20-30%)	
Markers	Graded p53/Ki-67, peripherally positive	Diffuse p53/Ki-67, high mitotic rate
Treatment	Excision	Excision or Mohs, with margin control
	Methotrexate/5-FU for select cases	
Recurrence/metastasis	Low (<2%), mainly local	Higher

From a diagnostic standpoint, shave or punch biopsies risk under‑sampling the pushing base and misclassifying keratoacanthoma as benign hyperkeratosis; therefore, full‑thickness excision remains the diagnostic gold standard when feasible [6]. When morphology is equivocal, immunohistochemistry (p16, p53, Ki‑67) and emerging molecular panels can aid differentiation: aberrant p16/p53 with a diffuse Ki‑67 pattern favors cSCC, whereas solitary keratoacanthoma more often shows a peripheral, graded Ki‑67 pattern [5]. For solitary extremity lesions, wide local excision with 3-5 mm margins achieves cure rates exceeding 95% [6]. Mohs micrographic surgery offers maximal tissue conservation and margin control for tumors on cosmetically sensitive sites or for recurrences [7]. Intralesional MTX (up to 25 mg per session) or 5‑FU can provide cytoreduction or definitive therapy in poor surgical candidates; in aggregated data, MTX yielded 88% clearance alone and 100% when followed by delayed excision [7,8].

Although metastatic risk is low (<2%), late recurrences have been documented; thus, dermatologic review every 6-12 months for two years is advised [2,6,9]. Our patient's outdoor occupation and fair skin confer continued risk for UV‑induced neoplasms, warranting ongoing photoprotection counseling. Limitations of this report include its single‑case design and short follow‑up; immunohistochemistry and molecular profiling were not performed, and longer observation is required to exclude delayed recurrence.

## Conclusions

Rapidly enlarging crateriform lesions on sun‑exposed skin should raise suspicion for keratoacanthoma‑type cSCC. Prompt full‑thickness excision provides simultaneous diagnosis and treatment, and the classical histopathology in this case, cup‑shaped architecture with a central keratin plug and a broad pushing base, confirmed the diagnosis. Our patient's uncomplicated healing and disease‑free status at three months are consistent with the excellent outcomes typically seen after standard excision with 3-5 mm margins for solitary extremity lesions. At the same time, because crateriform lesions can occasionally behave aggressively or recur, structured follow‑up remains prudent. Limitations of this report include its single‑patient design and short follow‑up, which preclude precise estimation of recurrence risk; longer observation will be necessary to confirm durable control.
